# Transferability of patients for radiation treatment between unmatched machines

**DOI:** 10.1002/acm2.13544

**Published:** 2022-01-30

**Authors:** Joseph J. Foy, Serpil K. Dogan, Poonam Yadav, Bharat B. Mittal, Indra J. Das

**Affiliations:** ^1^ Department of Radiation Oncology Northwestern Memorial Hospital Northwestern University Feinberg School of Medicine Chicago Illinois USA

**Keywords:** beam matching, operation, patient‐specific QA, treatment planning

## Abstract

**Purpose:**

The feasibility of transferring patients between unmatched machines for a limited number of treatment fractions was investigated for three‐dimensional conformal radiation therapy (3DCRT) and volumetric modulated arc therapy (VMAT) treatments.

**Methods:**

Eighty patient‐plans were evaluated on two unmatched linacs: Elekta Versa HD and Elekta Infinity. Plans were equally divided into pelvis 3DCRT, prostate VMAT, brain VMAT, and lung VMAT plans. While maintaining the number of monitor units (MUs), plans were recalculated on the machine not originally used for treatment. Relative differences in dose were calculated between machines for the target volume and organs at risk (OARs). Differences in mean dose were assessed with paired *t*‐tests (*p* < 0.05). The number of interchangeable fractions allowable before surpassing a cumulative ±5% difference in dose was determined. Additionally, patient‐specific quality assurance (PSQA) measurements using ArcCHECK for both machines were compared with distributions calculated on the machine originally used for treatment using gradient compensation (GC) with 2%/2‐mm criteria.

**Results:**

Interchanging the two machines for pelvic 3DCRT and VMAT (prostate, brain, and lung) plans resulted in an average change in target mean dose of 0.9%, −0.5%, 0.6%, 0.5%, respectively. Based on the differences in dose to the prescription point when changing machines, statistically, nearly one‐fourth of the prescribed fractions could be transferred between linacs for 3DCRT plans. While all of the prescribed fractions could typically be transferred among prostate VMAT plans, a rather large number of treatment fractions, 31% and 38%, could be transferred among brain and lung VMAT plans, respectively, without exceeding a ±5% change in the prescribed dose for two Elekta machines. Additionally, the OAR dosage was not affected within the given criterion with change of machine.

**Conclusions:**

Despite small differences in calculated dose, transferring patients between two unmatched Elekta machines with similar multileaf collimator (MLC)‐head for target coverage and minimum changes in OAR dose is possible for a limited number of fractions (≤3) to improve clinical flexibility and institutional throughput along with patient satisfaction. A similar study could be carried out for other machines for operational throughput.

## 1 INTRODUCTION

Accuracy, reliability, and patient satisfaction are hallmarks of most healthcare systems where each institution attempts to maintain a high degree of flexibility to adapt to unforeseen treatment issues. In radiation oncology, where treatment machines can differ in terms of model, beam energy, and multileaf collimator (MLC) design, transferring patients between linacs when a machine is down may prove difficult due to inherent differences in linac operation. Additionally, these issues may become more difficult to accommodate as machines age and unknown differences between linacs exasperate. Patient treatments are often postponed due to machine issues, creating unsatisfactory operational conditions and possibly poor patient satisfaction. Vendors attempt to provide 100% uptime by providing timely maintenance to their machines; however, machine uptime is more often limited to 97% during clinic operation.^1^, ^2^, ^3^, ^4^ Additionally, the machine uptime can vary based on how the uptime is defined and quantified as described in the literature.^5^

In high‐throughput clinics, transferring patient treatments among available machines can allow for increased flexibility and convenience; however, these alterations require accurately matched beam characteristics and dose delivery among the machines. Sjöström et al.^6^ published beam‐matching accuracy for 8 Varian (Varian Medical Systems, Palo Alto, CA) IX machines at a single institution. In general, the beam parameters were within ±2%, but showed significant differences (±5%) for 60 degree dynamic wedge. Modern accelerators such as the Varian TrueBeam can be tuned for a perfect match in terms of beam characteristic and delivery mechanism to within <0.5% as described by Glide‐Hurst et al.^7^ There has been much debate about linac beam‐matching issues as noted in TG‐106.^8^ Linac beam‐matching process is also elaborated on in the literature for three‐dimensional conformal radiation therapy (3DCRT) and intensity‐modulated radiation therapy (IMRT) for the ease of patient transferability.^6^, ^9^, ^10^, ^11^

Significant questions still need to be answered for beam matching and patient transferability, as it pertains to Elekta linacs (Elekta AB, Stockholm, Sweden). These include changes in dose to organs at risk (OARs), variations in target coverage, the dosimetrically tolerable number of transferred treatment fractions, and criteria for patient‐specific quality assurance (PSQA) for pretreatment plan transfer.

The purpose of this study was therefore to assess these issues and quantify the potential errors in delivered dose when transferring patients with unmatched machines. Using Elekta linacs with Agility heads, this study provides a recommended methodology when faced with the challenge of transferring patients between linacs in emergent situations. Evaluations were based on analysis of treatment plans consisting of 3DCRT, as well as prostate, brain, and lung volumetric modulated arc therapy (VMAT) plans with unmatched beams for patient treatment.

## 2 METHODS AND MATERIALS

**FIGURE 1:**
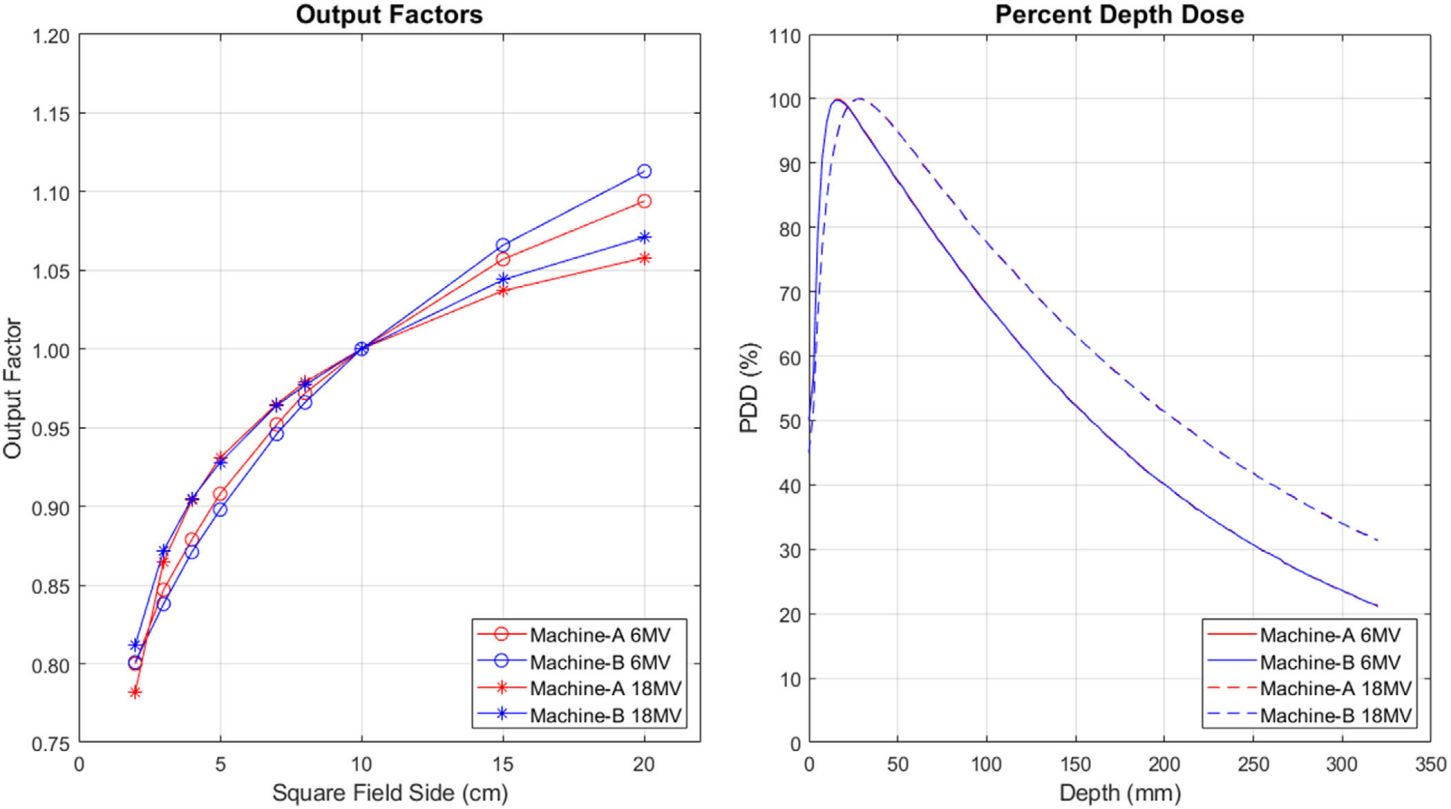
Output factors (left) and percent depth dose (PDD) curves (right) for 6 MV and 18 MV photon beams for machine‐A and machine‐B for square field. PDDs are shown for a 10 × 10 cm^2^ square field and are identical (<0.5%) for two machines, but the output factors are different

Two unmatched Elekta linacs in our department were evaluated for patient transferability in the case where one machine is unexpectedly out of commission: an Elekta Infinity (11‐year old), referred to here as “machine‐A” and an Elekta Versa HD (6‐year old), referred to as “machine‐B.” Both linacs are equipped with an Elekta Agility MLC. The Agility collimator is a binary MLC design composed of 160 leaves (80 leaf pairs), with a 5‐mm width at isocenter providing a maximum field size of 40 × 40 cm^2^. Agility leaves are not fitted with a tongue and groove but are slanted to minimize interleaf leakage. The average interleaf transmission is <0.5%, and the average transmission through the leaves is <0.38%, differing from older units whose characteristics have been compared with other MLCs.^12^ Both machines were commissioned separately in accordance with AAPM TG‐106,^8^ with MLC positioning tolerance of 1 mm as specified by AAPM TG‐142.^13^ Additionally daily, monthly, and annual calibrations are performed for both machines. Dosimetric calibrations are performed based on TG‐51^14^ and maintained within ±2% institutional criterion. Additionally, beam modeling for either linac was performed separately using independently acquired output factors and beam characteristics. Output calibration is performed at least each month to ensure linac output (*S*_cp_) is within 2% of baseline: 1 cGy/monitor unit (MU) under calibration conditions. Output factors for field sizes ranging from 2 × 2 to 20 × 20 cm^2^ for both machines are shown in Figure 1 along with the percent depth dose (PDD) curves for a 10 × 10 cm^2^ square field. The differences in PDD for both energies were within 0.5% and not reflected in Figure 1. Similarly, as both machines used Agility MLC head, the profiles are nearly identical (<0.5%) across all the fields.

For this study, 80 clinical treatment plans were obtained including 20 3DCRT pelvis plans, 20 prostate VMAT plans, 20 brain VMAT plans, and 20 lung VMAT plans. The 3DCRT pelvis plans, prostate VMAT plans, and lung VMAT plans were originally treated on machine‐A, while brain VMAT plans were originally treated on machine‐B. Each patient included in the present study was simulated on Philips Brilliance CT (Philips Healthcare, Cleveland, OH) in head‐first supine position with the slice thickness of 3 mm among pelvis, prostate, and lung plans, whereas brain VMAT plans were simulated with a 2‐mm slice thickness. All plans used the same HU to electron density table curve for planning. Details about the treatment sites, beam energy, OARs, and treatment planning techniques are provided in Table 1. Dose distributions were calculated using the adaptive convolution dose calculation algorithm and a 3‐mm grid resolution from a Pinnacle treatment planning system (TPS) V9.10 (Philips Radiation Oncology Systems, Fitchburg, WI). Pelvis 3DCRT plans used 18 MV photon beams, while all VMAT plans used 6 MV photon beams.

**TABLE 1:** Dosimetric characteristics among three‐dimensional conformal radiation therapy (3DCRT) and volumetric modulated arc therapy (VMAT) plans

Treatment site	Median dose per fraction (cGy) [range]	Median number of fractions [range]	Treatment technique	Beam energy (MV)	Organs at risk
Pelvis	180 [180–180]	25 [23–25]	3DCRT	18	Rectum, right femur, left femur, bladder
Prostate	180 [180–200]	3 [1–14]	VMAT	6	Rectum, right femur, left femur, bladder
Brain	200 [180–267]	23 [8–30]	VMAT	6	Brainstem, right lens, left lens, optic chiasm
Lung	200 [150–500]	30 [4–33]	VMAT	6	Spinal cord, ipsilateral lung, total lung, heart

Dosimetric parameters, such as mean and maximum dose to planning target volume (PTV), as well as the dose received by 95% of the PTV (D_95_), were recorded. The dose delivered to the prescription point, or the point to which Pinnacle aims to deliver the prescribed dose, was also recorded. For each treatment modality, four OARs consistent across all patients were assessed, and the mean dose to each was recorded. OARs are shown for each treatment site in Table 1. While maintaining the number of MUs, the treatment machine was changed from machine‐A to machine‐B or from machine‐B to machine‐A, and the dose distributions were recalculated on the linac not originally used for treatment. After the calculation was completed, the dosimetric parameters previously described were recorded. Differences in dose were assessed with a paired *t*‐test after correcting for the multiple comparisons across eight dosimetric parameters using Bonferroni (*p* < 0.006). Treatment planning objectives for each treatment modality used in this study are shown in Table 2. To assess differences in delivered dose distributions between two machines, PSQA was performed for all plans using ArcCHECK (Sun Nuclear Corporation, Melbourne, FL).

**TABLE 2:** Dose objectives used during planning of each treatment site

Pelvis and prostate		Brain		Lung	
Structure	Dose constraint	Structure	Dose constraint	Structure	Dose constraint
Rectum	*V*_75_ <15%	Brainstem	Max ≤54 Gy	Esophagus	*V*_50_ <32%
	*V*_70_ <20%		*V*_59_ <10 cm^3^		*V*_60_ <33%
	*V*_65_ <25%	Lens	Max <7 Gy	Ipsilateral lung	*V*_20_ <15%
	*V*_60_ <35%	Chiasm	Max <55 Gy		*V*_10_ <35%
	*V*_50_ <50%				*V*_5_ <65%
Bladder	*V*_80_ <15%			Cont. lung	*V*_5_ <10%
	*V*_75_ <25%			Heart	Mean <1 Gy
	*V*_70_ <35%				*V*_20_ <5%
	*V*_65_ <50%				
Femoral head	*V*_50_ <10%				

Using the dose grids created on the linac originally used for treatment, measured dose distributions were compared to calculated dose grids with gradient compensation (GC).^15^ A 20% dose threshold and the 2%/2 mm institutional criterion with 90% passing rates were utilized for our PSQA, even though TG218^16^ uses 3%/2 mm, 90% passing rate criterion with 10% threshold. In other words, treatment plans originally delivered on machine‐A were delivered on both machine‐A and machine‐B, but the measured dose distributions were both compared to the dose grids calculated on machine‐A and the same is true for patients originally treated on machine‐B.

Similar to gamma analysis, GC compares the dose difference and distance to agreement (DTA) for each point between the measured and calculated dose distributions; however, GC adjusts the dose difference by the product of the local dose gradient and a user‐defined uncertainty perimeter.

Managing machine issues may require patients to be moved between machines. Therefore, the number of allowable fractions that can be transferred between unmatched linacs was also investigated and the methodology presented for clinicians to implement for any set of linacs. ICRU‐50 states that while more homogeneous dose distributions are typically desirable, a degree of heterogeneity is expected. Therefore, the delivered dose should be within −5% and +7% of the prescribed dose.^17^ Considering these recommendations, the number of transferable fractions was determined based on the relative difference in calculated dose to the prescription point between linacs while maintaining the number of MUs. The number of transferable fractions was calculated as the ratio of the 5% allowable dose difference and the relative dose difference to the prescription point due to the change in treatment machine. Based on the number of fractions prescribed for a patient's intended treatment (Table 1), the relative number of transferable fractions was also calculated.

## 3 RESULTS

Four‐field 3DCRT, prostate VMAT, brain VMAT, and lung VMAT plans (20 plans each) were computed on two unmatched linacs while maintaining beam weighting and the total number of MUs.

**FIGURE 2:**
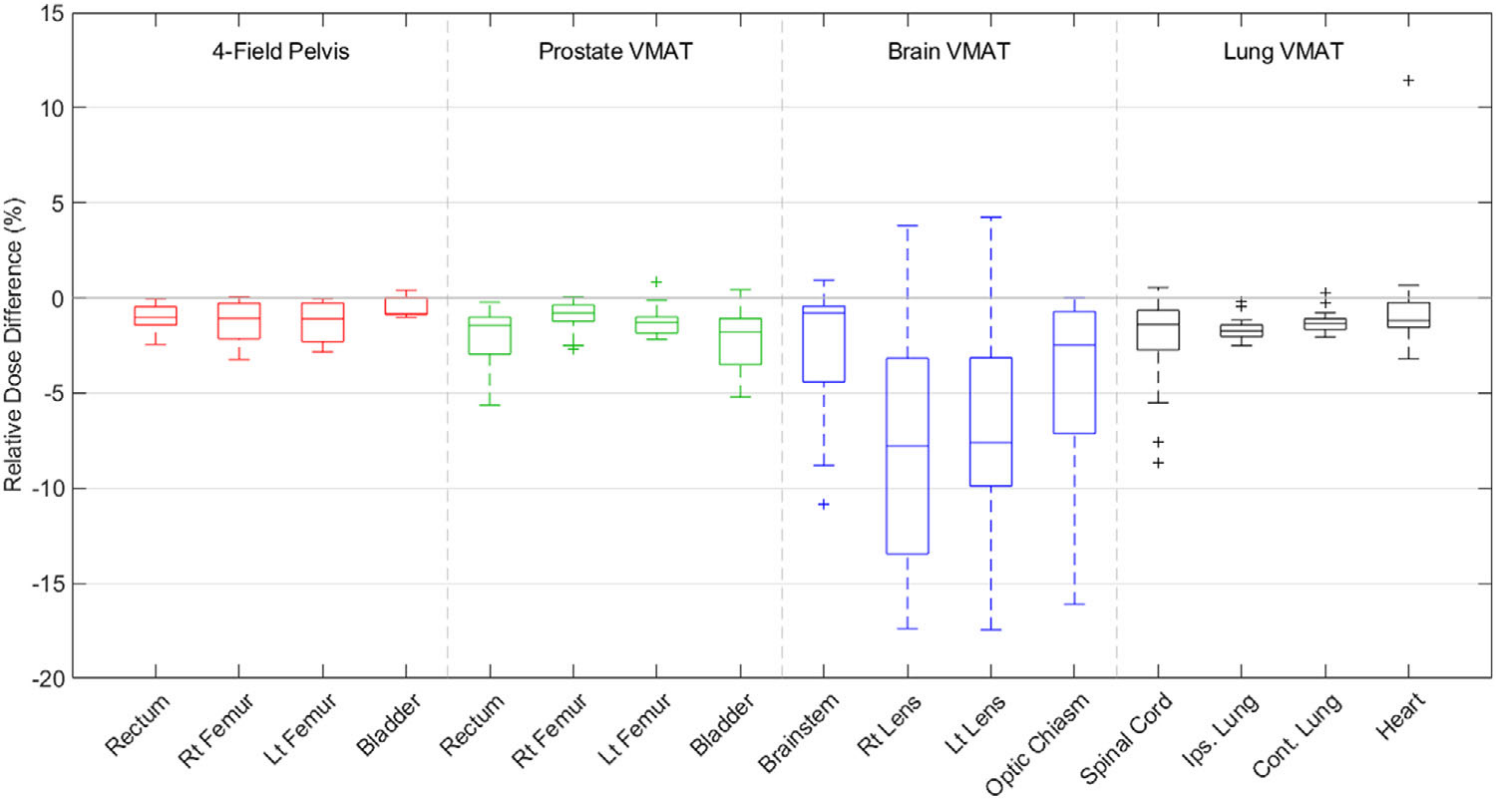
Boxplots reflecting the relative difference in mean dose to the organs at risk (OARs) among three‐dimensional conformal radiation therapy (3DCRT) and volumetric modulated arc therapy (VMAT) plans. Values less than zero indicate an increased dose when calculated on machine‐B as opposed to machine‐A. Boxes extend to the first and third quartile, with outliers represented by + sign

The relative differences in mean dose to each of the four OARs among 3DCRT and VMAT plans are shown in Figure 2. For 3DCRT pelvis plans, all patients received an increased dose to all OARs when calculated on machine‐B besides the right femur and bladder, which resulted in two and five patients receiving reduced dose, respectively. Similar results are reported among any of the VMAT plans with at most four patients receiving increased dose to the OARs when treated on machine‐A. As shown in Figure 2, brain VMAT plans resulted in the greatest variability in relative dose differences among patients with standard deviations of 3.4%, 6.2%, 5.9%, and 4.3% for the brainstem, right lens, left lens, and optic chiasm, respectively. The mean dose reflected significant differences between machines among all OARs for each treatment site.

**FIGURE 3:**
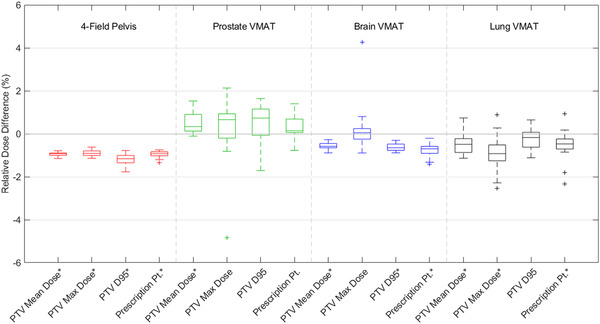
Boxplots reflecting the relative difference in dose to the planned target volume (PTV) and prescription point for three‐dimensional conformal radiation therapy (3DCRT) and volumetric modulated arc therapy (VMAT) plans. Values less than zero indicate an increased dose when calculated on machine‐B as opposed to machine‐A. Boxes extend to the first and third quartile, with outliers represented by + symbol. Dosimetric parameters reflecting significant differences between machines are indicated by an asterisk (*)

The relative differences in mean dose to the PTV, maximum dose to the PTV, the dose to 95% of the PTV volume (*D*_95_), and the dose to the prescription point are reported in Figure 3 for each treatment site. Among four‐field pelvis plans, the mean dose, maximum dose, *D*_95_, and the dose to the prescription points were greater when calculated on machine‐B compared to machine‐A. The VMAT plans reflected greater variability, with 90% of prostate VMAT plans reflecting a reduced mean dose to the PTV when calculated on machine‐B. In contrast, 100% and 90% of brain and lung VMAT plans resulted in an increased mean dose to the PTV when calculated on machine‐B, respectively. Compared to the mean dose to the OARs, the dose to the PTV and prescription point, as indicated in Figure 3, reflected reduced variability among patients with a range of relative differences in mean dose to the PTV of 0.36%, 1.64%, 0.62%, and 1.87% among four‐field pelvis, prostate VMAT, brain VMAT, and lung VMAT plans, respectively.

The dosimetric parameters in Figure 3 marked with an asterisk (*) reflected significant differences in dose between machine‐A and machine‐B illustrating the increased variability in dose differences to the PTV and prescription point relative to the OARs, where all structures reflected significant differences. While all four dosimetric parameters reflected significance among four‐field pelvis plans, only the mean PTV dose was significant among prostate VMAT plans. In addition, only the maximum PTV dose did not reflect significance among brain VMAT plans, whereas only the PTV *D*_95_ did not reflect significance for lung VMAT plans.

As shown in Figure 2, the lenses and optic chiasm reflect the greatest variation in relative dose differences among brain VMAT plans, whereas the ipsilateral lung and total lung volume reflected the smallest variability among lung VMAT plans. Relative differences in dose were not significantly correlated with structure volume for any OAR or PTV, except only the bladder for four‐field pelvis plans and the PTV for brain VMAT plans were different as reflected by Spearman's correlation coefficients greater than 0.3 (*ρ* = 0.33 and 0.61, respectively).

The dose to the prescription point showed similar trends to that of the mean PTV dose as noted in Figure 3: all four‐field pelvis and brain VMAT plans resulted in an increased dose to the prescription point when treated on machine‐B, whereas 90% of lung VMAT plans reflected an increased dose. In comparison, 80% of prostate VMAT plans resulted in a reduced dose to the prescription point when calculated on machine‐B.

**FIGURE 4:**
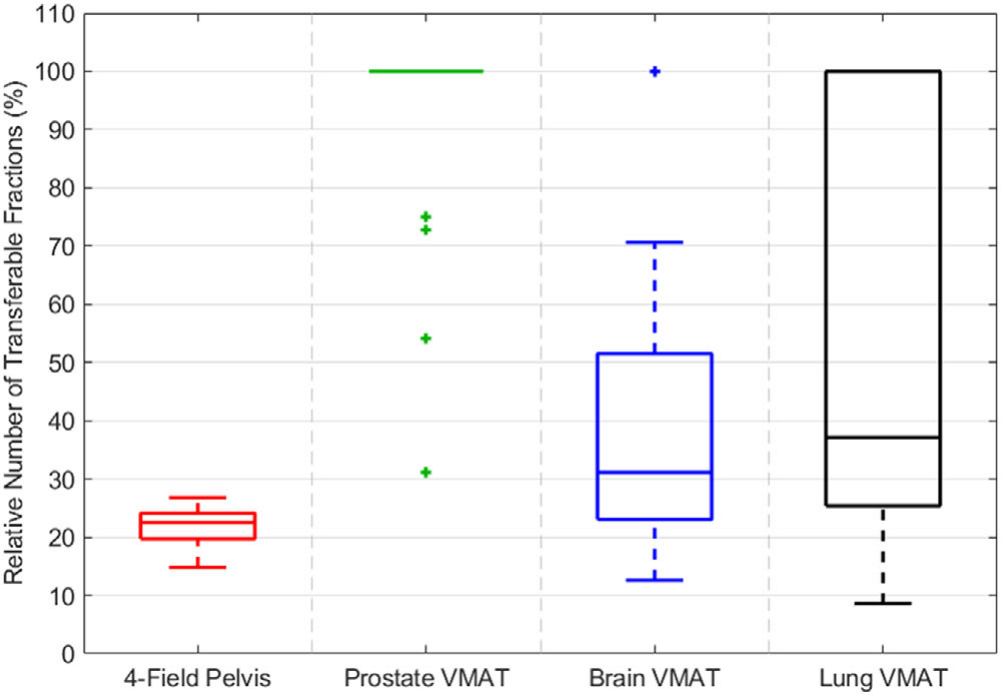
Boxplots illustrating the relative number of transferable fractions between unmatched machines among four‐field pelvis and volumetric modulated arc therapy (VMAT) plans

Based on the relative difference in dose to the prescription point, the relative number of allowable fractions that can be transferred between machine‐A and machine‐B was calculated and is shown in Figure 4. For 3DCRT plans, the mean number of transferable fractions between machine‐A and machine‐B was five fractions corresponding to 22.5% of the prescribed number of fractions (range: 14.8%–26.8%). In comparison, prostate VMAT plans resulted in an average of nine transferable fractions; however, prostate VMAT plans were consistently prescribed fewer fractions than 3DCRT plans (Table 1). Consequently, prostate VMAT plans resulted in the greatest relative number of transferable fractions, with 80% of patients allowing the entire treatment to be transferred without deviating from the prescribed dose by more than 5%; however, 3DCRT and prostate VMAT plans utilized different photon beam energies and output factors, which will also impact the changes in dose when transferring patients between linacs. Brain and lung VMAT plans reflected much greater variability among the relative number of transferable fractions, because the number of prescribed fractions was much more variable for these plans. While prostate VMAT plans had a maximum of 14 prescribed treatment fractions, brain VMAT plans ranged from eight to 30 treatment fractions, and lung VMAT plans ranged from four to 33 treatment fractions.^17^ Out of all 3DCRT and VMAT plans, the minimum number of transferable fractions was number.

**FIGURE 5:**
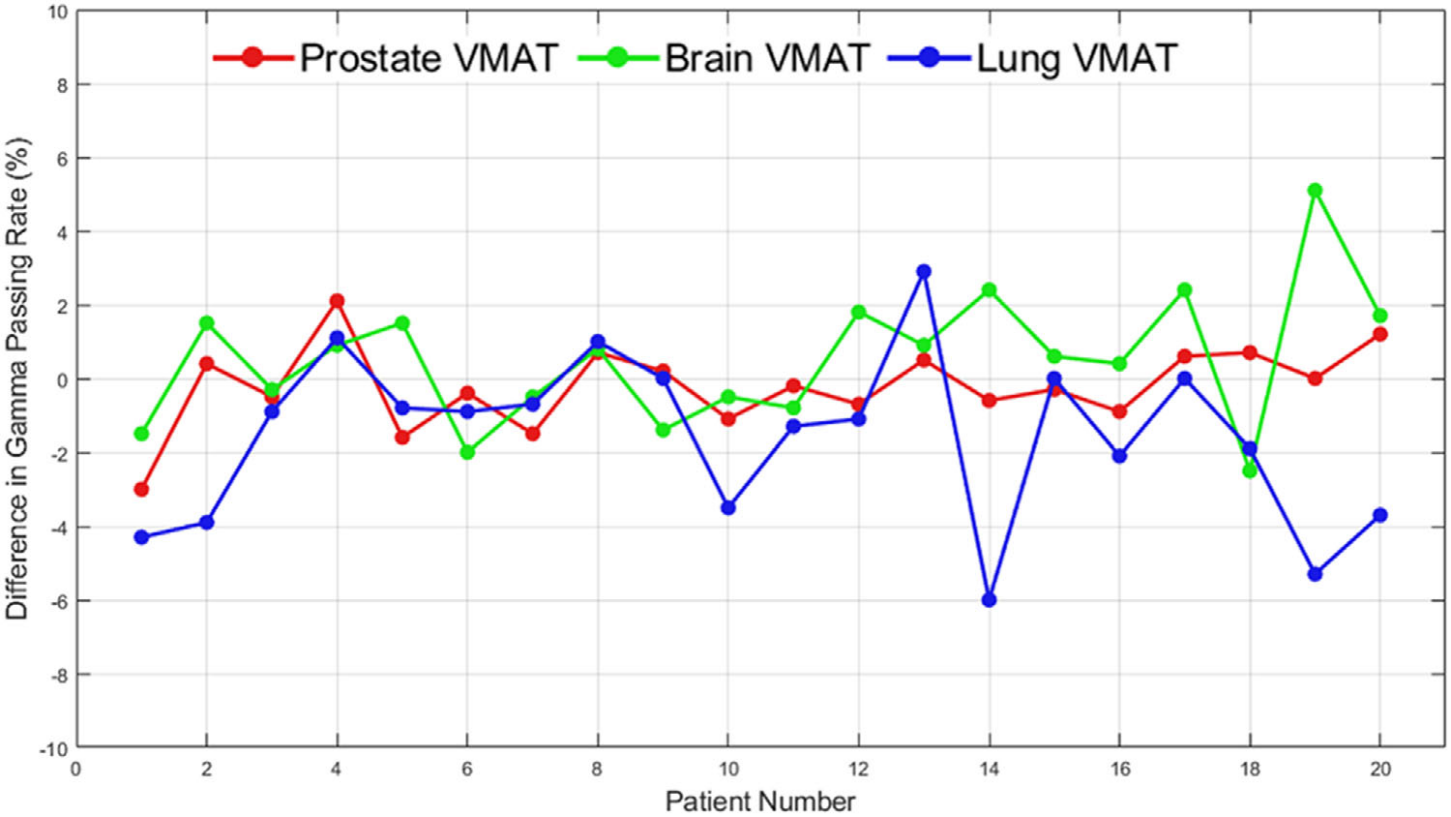
Differences in gradient compensation (GC) passing rates among prostate, brain, and lung volumetric modulated arc therapy (VMAT) plans when measured with ArcCHECK. Passing rates were assessed with a 2.0%/2.0 mm for 90% threshold criterion

The VMAT plans were assessed after measurement using ArcCHECK on both machine‐A and machine‐B and compared with the planned dose distributions calculated on the machine originally used for treatment. All VMAT plans resulted in a GC passing rate reflecting clinical quality (>90%) when delivered and measured on either machine‐A or machine‐B. The differences in GC passing rates using a 2.0%/2.0 mm threshold when delivered on both machines are shown in Figure 5 for prostate, brain, and lung VMAT plans. Negative values indicate that machine‐A had a lower passing rate than machine‐B, while positive differences indicate that machine‐A had a higher passing rate than machine‐B. Among prostate, brain, and lung VMAT plans, the mean difference in the GC passing rates were 0.22% (range: −3.0% to 2.1%), 0.53% (range: −2.5% to 5.1%), and −1.57% (range: −6.0% to 2.9%), respectively. Differences in GC passing rates reflected no correlation (|*r*| < 0.4) with differences in mean dose to the PTV and the prescription point among prostate, brain, and lung VMAT plans.

## 4 DISCUSSION

During a course of radiotherapy, machine issues or scheduling conflicts often arise that may delay or extend the planned duration of the treatments. Therefore, flexible patient scheduling and the ability to transfer patient treatment plans between machines can be helpful in avoiding interruption in the patient's prescribed course of treatment. The presented investigation aimed to assess the dosimetric effects of transferring patients between two unmatched linacs as well as to develop a methodology to determine the number of treatment fractions that could be transferred without violating the ICRU‐50 criterion.^17^ Additionally, for a mixed machine department, a similar study needs to be conducted to evaluate the applicability and transferability issues.

The results presented here for Elekta machine show that for 3DCRT plans in pelvis patients, the doses to the rectum, left femur, PTV, and prescription point were greater when calculated on machine‐B for all patients than when calculated on machine‐A, as shown in Figure 2. For the right femur and bladder, 90% and 75% of plans resulted in greater mean dose when calculated on machine‐B, respectively. Despite these systematic differences, the average relative dose differences between linacs were less than 1.3% for all six structures. The consistent differences in calculated doses to each tissue can be attributed to the higher output factors between the two linacs, as shown in Figure 1. The 3DCRT plans were created with four fields having field sizes on the order of 20 × 20 cm^2^ corresponding to a 1.2% increase in the output factor when moving from machine‐A to machine‐B and consequently an increase in dose. These results indicate that while individual machines may consistently deliver different doses to various tissues, these differences may still be permissible based on ICRU‐50 and should be evaluated on a case‐by‐case basis. Additionally, the variability of the relative dose differences was greater among OARs than for the PTV and prescription point, indicating that the doses to tissues outside the confines of the target are expected to reflect a greater dependency on the particular linac used for treatment. While the doses to tissues within the treatment fields are primarily dependent on the machine output, out‐of‐field doses have greater uncertainty due to differences in head scatter, transmission, and dose profiles.

Similar results were found for VMAT prostate plans: the dose to OARs was greater when calculated on machine‐B for the majority of patients and reflected the significant variability in relative dose difference among patients. In addition, the relative dose difference and the variability in the relative dose difference were lower for the PTV and prescription point. In contrast to the 3DCRT plans, the mean dose to the PTV and prescription point was reduced when calculated on machine‐B for 90% and 80% of patients, respectively. This difference can be attributed to the differences in photon energy and output factors utilized in prostate VMAT plans. While the output factors for the larger field sizes used during 3DCRT treatments are greater for machine‐B, the output factors for 6 MV photon beams and smaller field sizes (<10 × 10 cm^2^) commonly used during prostate VMAT treatments are consistently greater by as much as 1.1% compared to machine‐A.

While prostate VMAT plans reflected a reduction in dose to the PTV and prescription point when treated on machine‐B, brain and lung VMAT plans resulted in similar results to those of the 3DCRT pelvis plans: increased mean doses to OARs, PTV, and prescription point for most plans. However, brain and lung VMAT plans include different tissues and inhomogeneities than prostate VMAT plans. While targets and OARs are typically surrounded by a relatively homogeneous medium, treatment plans of the brain are often complicated by the surrounding sinuses and bony anatomy. Similarly, lung plans are complicated by the surrounding air and stark differences in density between the lung volume and soft tissue. These differences may increase the uncertainty and variability in the dose calculation when patients are transferred between unmatched linacs.

ICRU 50^17^ indicates that the dose to the prescription point must remain within ±5% of the intended prescription dose to transfer patients between unmatched linacs. The large degree of variability in the number of transferable fractions among prostate, brain, and lung VMAT plans indicates that each patient plan must be evaluated individually to assess the effect on the delivered dose. Nevertheless, some patients may be transferred between machines for the entirety of their treatment without greatly affecting the delivered dose, particularly for prostate VMAT plans where tissue inhomogeneities are minimized. However, all patients could be transferred for at least three fractions, which would typically allow for any required maintenance to be completed to make the original linac operational. Additionally, the number of transferable fractions may differ depending on the machines that are interchanged. For example, one prostate VMAT patient indicated a 0.8% reduction in dose to the prescription point if all 14 of the prescribed fractions were transferred from machine‐A to machine‐B. At the same time, the dose to the rectum would be 39.5% higher, which may not be permissible if the dose to the rectum was already approaching the dose constraints in the original plan. Although, if the original plan was calculated on machine‐B and transferred to machine‐A, one may expect to find the opposite results: the dose to the prescription point will slightly increase, while the dose to the OARs is reduced. In this case, the dose to the OARs would not be a limiting factor, and transferring the patient may be a viable option. While the dose to the prescription point may be relatively unchanged due to changing the treatment machine, OARs show greater variability in the relative dose differences, and the doses to all OARs reflecting significant differences between linacs. These results indicate that changes in dose to the OARs must be assessed when transferring patients between unmatched linacs.

The 3DCRT plans reflected much less variability among patients in the number of transferable fractions: each patient could be transferred between machines for 14.8%–26.8% of the prescribed fractions (corresponding to four to seven fractions). Among prostate, brain, and lung VMAT plans, patients could be transferred for at least 31.8%, 12.6%, and 8.6% of the total prescribed fractions, respectively; however, each treatment site among VMAT plans allowed for all of the prescribed fractions to be transferred between linacs for at least one patient (Figure 4). While the number of transferable fractions is increased for VMAT plans, VMAT plans are typically treated with a fewer fractions resulting in an increase in the relative number of transferable fractions. One should also note that because of the differences in beam energies used for 3DCRT and VMAT plans, the output factors must also be considered. These results indicate that the relative number of transferable fractions is highly dependent on several factors, including the treatment modality, beam energy, dose constraints, and the number of prescribed fractions. Therefore, clinicians should evaluate the dosimetric impact for each patient to be transferred individually by calculating the difference in dose to each OAR, the target, and prescription point when the linac is changed but the MUs are held constant. The number of transferable fractions is then calculated as the 5% dose different threshold divided by the relative difference in dose to the prescription point due to the changing treatment machines. This flexibility may allow clinics adequate time for linac maintenance without disturbing a patient's intended treatment course; however, these results are contingent on the limiting factors such as dose to normal tissues and must be assessed for each patient.

The PSQA results shown in Figure 5 provide confidence in the accuracy of the planned and the delivered doses in case a patient's treatment plan is transferred between machine‐A and machine‐B. All VMAT plans surpassed the 90% GC passing rate criteria used clinically when delivered on either machine. One brain VMAT and one lung VMAT patient achieved a 90% passing rate when measured on the machine originally used for treatment; however, when the plan was measured on the opposite machine, the GC passing rate increased by more than 5% for both patients. Therefore, one may not necessarily expect to observe a poorer agreement between planned and delivered dose distributions when treating patients on another machine. This claim is further strengthened by the lack of correlation between the differences in the GC passing rates and the differences in the mean dose to the PTV and prescription point. Based on random day‐to‐day fluctuations in output, the calculated dose distribution may be in better or poorer agreement with the measured dose distribution, which should also be accounted for during PSQA. When delivered on machine‐B, the PTV and prescription point received an increase dose for most patients among prostate VMAT plans. In contrast, brain and lung VMAT plans resulted in a reduced dose to the PTV and prescription point for most patients. These trends, however, were not realized in the agreement between calculated and measured dose distributions. As shown in Figure 5, the relative differences in GC passing rates are centered on 0% with no significant bias in whether the changing of a machine would increase or decrease the GC passing rate. Changing VMAT plans from machine‐A to machine‐B would result in an expected average decrease in the GC passing rate of 0.53% among brain VMAT plans and an average increase in the GC passing rate of 0.22% and 1.57% among prostate and lung VMAT plans, respectively. If GC passing rates were seen to decrease below an acceptable threshold (i.e., 90%), these differences may still be allowable and accounted for by adjusting the already conservative 2.0%/2.0 mm criterion implemented in this study.

While the results reported here suggest that patients undergoing treatment using four‐field 3DCRT and VMAT plans for prostate, brain, and lung cancer may be transferred for at least three of the prescribed treatment fractions, it should be emphasized that these values pertain only to the two linacs analyzed in this study. When patients are transferred between any combinations of linacs, clinicians should implement the methodology described here to determine the number of transferred fractions permissible without exceeding a ±5% change in the prescription dose while also considering changes in dose to the normal tissues. At the same time, clinics would typically not consider transferring patients between linacs with remarkably different construction and dosimetric properties. Future studies will therefore analyze the changes in the number of transferable fractions for linacs with different manufacturers (e.g., Elekta and Varian), MLC configurations, and commissioning standards (e.g., conventional and stereotactic radiosurgery). Additional studies will investigate whether these findings translate to more complex plans with smaller field sizes such as stereotactic body radiation therapy (SBRT). Xu et al.^18^ investigated the difference in delivered doses and gamma passing rate when transferring highly modulated VMAT plans among beam‐matched linacs. They reported dose differences less than 0.1% for field larger than 10 × 10 cm^2^, whereas differences in dose approached 1.3% among linacs for field sizes on the order of 1 × 1 cm^2^. They also reported passing rates exceeding 90% for all plans delivered on each machine when using 2.0%/2.0 mm criterion, demonstrating that similar results may be found for unmatched linacs of similar construction; however, additional studies must be conducted to validate this claim. Relatively simple plans with a limited number of treatment parameters and established fractionation schemes, such as four‐field 3DCRT plans, result in little variability in the number of transferable fractions. In contrast, VMAT plans can potentially allow for many more transferable fractions. Therefore, more complex treatment modalities like SBRT may similarly allow for an increased number of fractions that can be treated on unmatched linacs.

## 5 CONCLUSIONS

Transferring patients between linacs may be necessary in the case of a machine malfunction to prevent disturbing a patient's intended course of treatment. For 3DCRT and VMAT prostate, brain, and lung plans, clinically unmatched Elekta linacs with the same MLC head (providing nearly identical PDD and profiles) demonstrated adequate agreement in the delivered doses to the PTV, OARs, and the prescription point. Differences in the dose to the prescription point indicated that patients can be transferred between two linacs for at least three treatment fractions; however, the differences in the dose due to this transfer should be assessed and documented for each patient. Additionally, calculated and measured dose distributions among VMAT plans reflected good agreement with no systematic differences in the GC passing rate between two machines. These findings may aid clinicians in the scheduling of patients and provide some flexibility in patient treatment when a transfer between unmatched linacs is necessary. While the values presented here may not apply to the general medical physics community with many combinations of different linacs, the methods outlined in this study could be implemented a priori to determine how many fractions can be transferred between unmatched linacs without significantly degrading treatment plan quality and institutional dosimetric criterion.

## CONFLICT OF INTEREST

The authors declare that there is no conflict of interest.

## AUTHOR CONTRIBUTIONS

All authors contributed equally to this manuscript.
